# Honoring Elders, Honoring Culture: Rethinking Rural Healthcare for Native Communities

**DOI:** 10.1093/geroni/igaf122.832

**Published:** 2025-12-31

**Authors:** Pamela Monaghan-Geernaert

**Affiliations:** Northern State University, Aberdeen, South Dakota, United States

## Abstract

Providing culturally competent care for Native American elders in rural communities requires an understanding of their history and culture. Many Indigenous elders prefer that the care they receive focus on community, spirituality, and traditional healing practices. These modalities of care often differ from mainstream Western healthcare approaches and are furthermore, not reimbursable expenses in nursing home and other health care environments. Additionally, it is unlikely that staff have been trained to manage, promote or administer this kind of culturally responsive care. Barriers such as lack of culturally trained staff, geographic isolation, and systemic healthcare disparities make it challenging to implement culturally responsive care in rural settings. This study explores how rural communities engage with Native elders to develop care models that honor specific tribal cultural practices while addressing medical needs. Through qualitative research with Native and non-Native caregivers, this presentation highlights best practices. Findings demonstrate that community-driven solutions, staff cultural training, and integration of Indigenous healing methods improve patient well-being and caregiver effectiveness. By recognizing and respecting Native elders’ preferences, healthcare providers in rural communities can create inclusive, culturally sustaining care environments that uphold dignity and cultural continuity.

